# Is thyroid dysfunction a cause or a trigger of bipolar disorder? a case report

**DOI:** 10.3389/fpsyt.2024.1473536

**Published:** 2024-09-12

**Authors:** Jiashu Yao, Jiating Xu, Rong Yan, Ruihuan Ye, Yuedi Shen, Ning Dai, Wei Chen

**Affiliations:** ^1^ Department of Psychiatry, Sir Run Run Shaw Hospital, School of Medcine, Zhejiang University, Hangzhou, Zhejiang, China; ^2^ The Second Department of General Psychiatry, The Third Hospital of Quzhou City, Quzhou, Zhejiang, China; ^3^ Department of Psychiatry, Shaoxing 7th People’s Hospital, Shaoxing, Zhejiang, China; ^4^ The Affiliated Hospital of Hangzhou Normal University, Hangzhou Normal University, Hangzhou, Zhejiang, China; ^5^ Department of Gastroenterology, Sir Run Run Shaw Hospital, School of Medcine, Zhejiang University, Hangzhou, Zhejiang, China

**Keywords:** hyperthyroidism, comorbidity, psychosomatic disorders, bipolar disorder, bipolar disorder due to another medical condition

## Abstract

Here we report on a case of a 40-year-old female patient who presented with hypomanic episode after hyperthyroidism and major depressive episode after hypothyroidism, which was initially misdiagnosed as bipolar disorder due to another medical condition, and was found to be a co-morbid bipolar disorder of hyperthyroidism after treatment and follow-up. The course of diagnosis and treatment in this case suggests a close temporal relationship does not necessarily mean that there is a causal relationship on a physiologic level. User’s Guide for the SCID-5-CV Structured Clinical Interview for DSM-5 Disorders elaborate that the diagnosis of “……due to another medical condition” is relatively rare, and co-morbidity between psychiatric disorders and somatic diseases is much more common. Therefore, the relationship between somatic diseases and psychiatric disorders requires careful study of symptom correlation and more time for observational follow-up. When in doubt, the examiner’s default assumption should be that the somatic disease is not the cause (i.e., the psychiatric disorder is primary).

## Introduction

The integrative concept of psychosomatic disorders (1) is increasingly emphasized in clinical practice. Somatic diseases can lead to psychiatric symptoms, such as adrenal insufficiency causing depressive symptoms (2), and psychiatric disorders can affect physical symptoms, like anxiety disorders exacerbating abdominal pain and diarrhea (3). When somatic disease and psychiatric disorder coexist, especially when the two systems producing the symptoms influence each other, clinicians often use monism to explain all symptoms. However, patients differ, and while we recognize psychosomatic integration, we must also consider the possibility that the relationship between somatic disease and psychiatric disorder is not as strong as assumed.

We report a case of a patient who presented with hypomanic episode after hyperthyroidism and major depressive episode after hypothyroidism, initially misdiagnosed as bipolar disorder due to another medical condition. After treatment and follow-up, it was found to be a co-morbid bipolar disorder with hyperthyroidism.

## History of present illness

A 40-year-old female patient presented 10 months ago with heat intolerance, oligomenorrhea, and a weight loss of 1-2 kg over 2 months. Her physical examination report showed hyperthyroidism 8 months ago but did not follow up in time. 7 months ago, she became very happy and energetic, sleeping 2 hours less than usual but still feeling energetic throughout the day. She was more active, went out with friends more often, talked more, and was more confident than usual. She spent more money and often bought unnecessary things. Her family noticed these changes and found her overexcited.

6 months ago, she was reminded by her family to visit the hospital, where she was diagnosed with hyperthyroidism and treated with methimazole tablets 20mg once daily. 4 months ago, her high mood stabilized, and her thyroid function returned to normal.

3 months ago, she developed worrying and overthinking, focusing on the negative, and had difficulty making decisions. She constantly rubbed her hands and thighs together, felt depressed, and wanted to cry. She lost interest in activities she used to enjoy, felt her brain was sluggish, had difficulty concentrating, and experienced memory loss. She has decreased appetite, constipation, fear of cold, easy fatigue. She denied suicidal thoughts.

Upon revisiting the endocrinology department, tests showed hypothyroidism, so the methimazole dose was gradually reduced to 5mg once daily. 1 month ago, her thyroid function was re-examined and found normal, but her mood remained depressive. The endocrinologist referred her to the psychiatry department, and she was hospitalized in our department 2 weeks ago.

## Medical history

Apart from hyperthyroidism, the patient denied any other physical illnesses, surgeries, trauma, or substance abuse. Previous annual physicals showed no thyroid function problems.

## Personal history

The patient has a Bachelor’s degree and is a college teacher. She was previously outgoing, enjoyed making friends, and was meticulous about hygiene. Since the onset of the COVID-19 pandemic, her cleanliness habits worsened. She frequently washed her hands, more than 10 times a day, and always did so after touching public places. Although her mother thought she was excessively clean, she did not think the symptoms bothered her too much. She is married, has a good relationship with her husband, and has a daughter. However, her husband disliked her cleanliness habits.

## Hospitalization treatment

On admission, the patient’s blood tests, blood biochemistry, thyroid function, cranial MRI, anti-syphilis spirochete antibody, HIV antibody, chest CT, and abdominal ultrasound were all negative. Her scores on the Hamilton Rating Scale for Depression (HAMD-17) were 25, Hamilton Anxiety Rating Scale (HAMA) were 18, Pittsburgh Sleep Quality Index (PSQI) were 12, Mood Disorder Questionnaire (MDQ) were 2, and Hypomania Symptom Checklist-32 (HCL-32) were 18.

The diagnosis of major depressive disorder due to another medical condition was considered, and her previous hypomanic symptoms were thought to be due to hyperthyroidism. Escitalopram was gradually increased to 20mg once daily. After two weeks of hospitalization, her depression and anxiety significantly decreased. After her symptoms almost disappeared (5 points for HAMD-17, 4 points for HAMA), she was discharged with normalized thyroid hormone levels.

## Follow-up after discharge

After discharge, the patient again exhibited symptoms of high emotion, high energy, and increased activity, scoring 14 on the Young’s Mania Rating Scale (YMRS). Her thyroid function was normal, so the diagnosis was changed to bipolar disorder. Her medication was adjusted to lithium carbonate 0.9 once daily and aripiprazole 10mg once nightly. Her mood stabilized after three weeks. Maintaining the same medications, she was followed up for 5 months with normal thyroid function and stable mood, with HAMD-17 and YMRS scores of 5, 6 (1 month later), 5, 4 (3 months later), and 6, 5 (5 months later), respectively.

## Discussion

**Figure 1:**
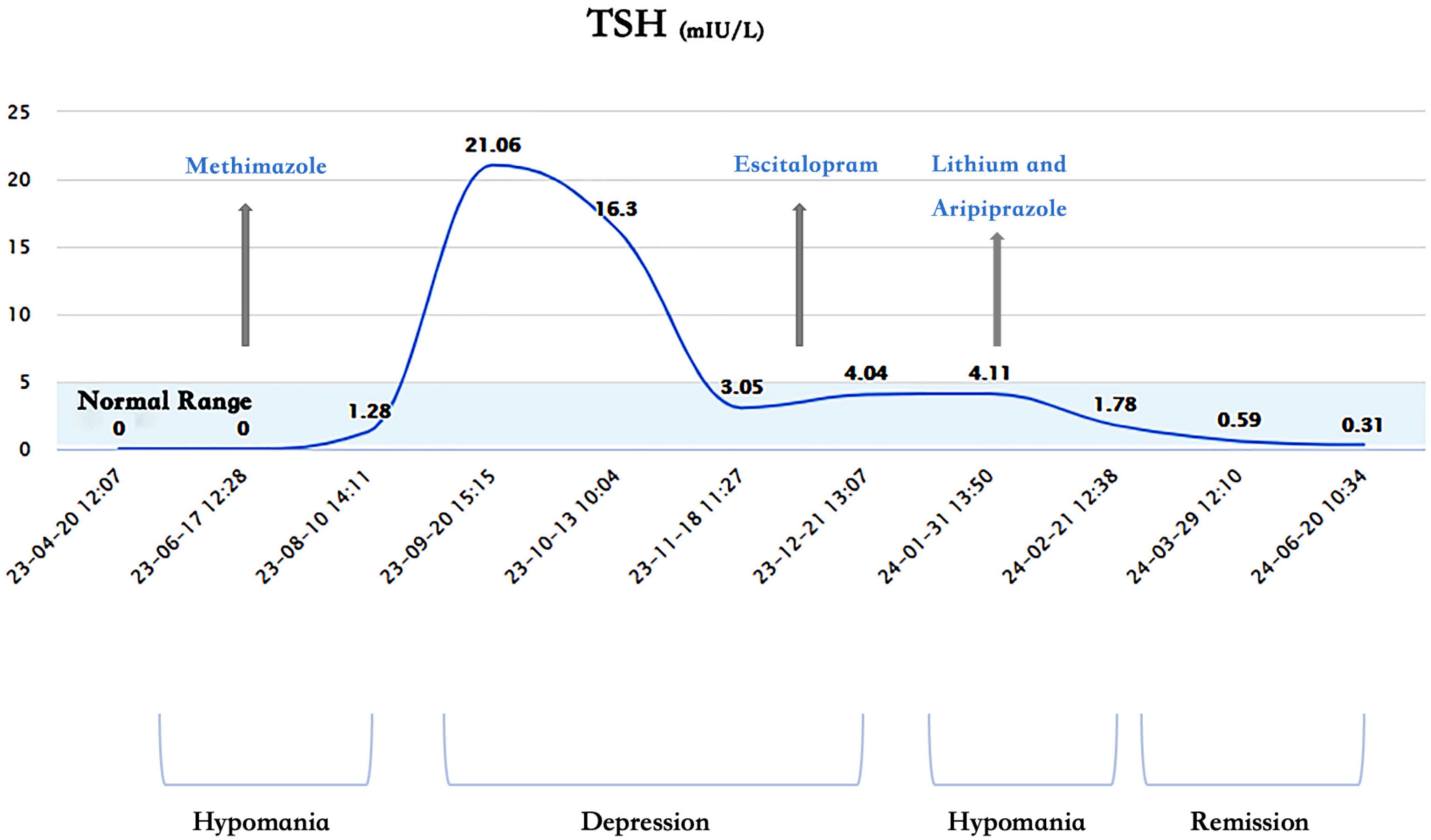
Changes of thyroid stimulating hormone (TSH) and mood.

**Figure 2:**
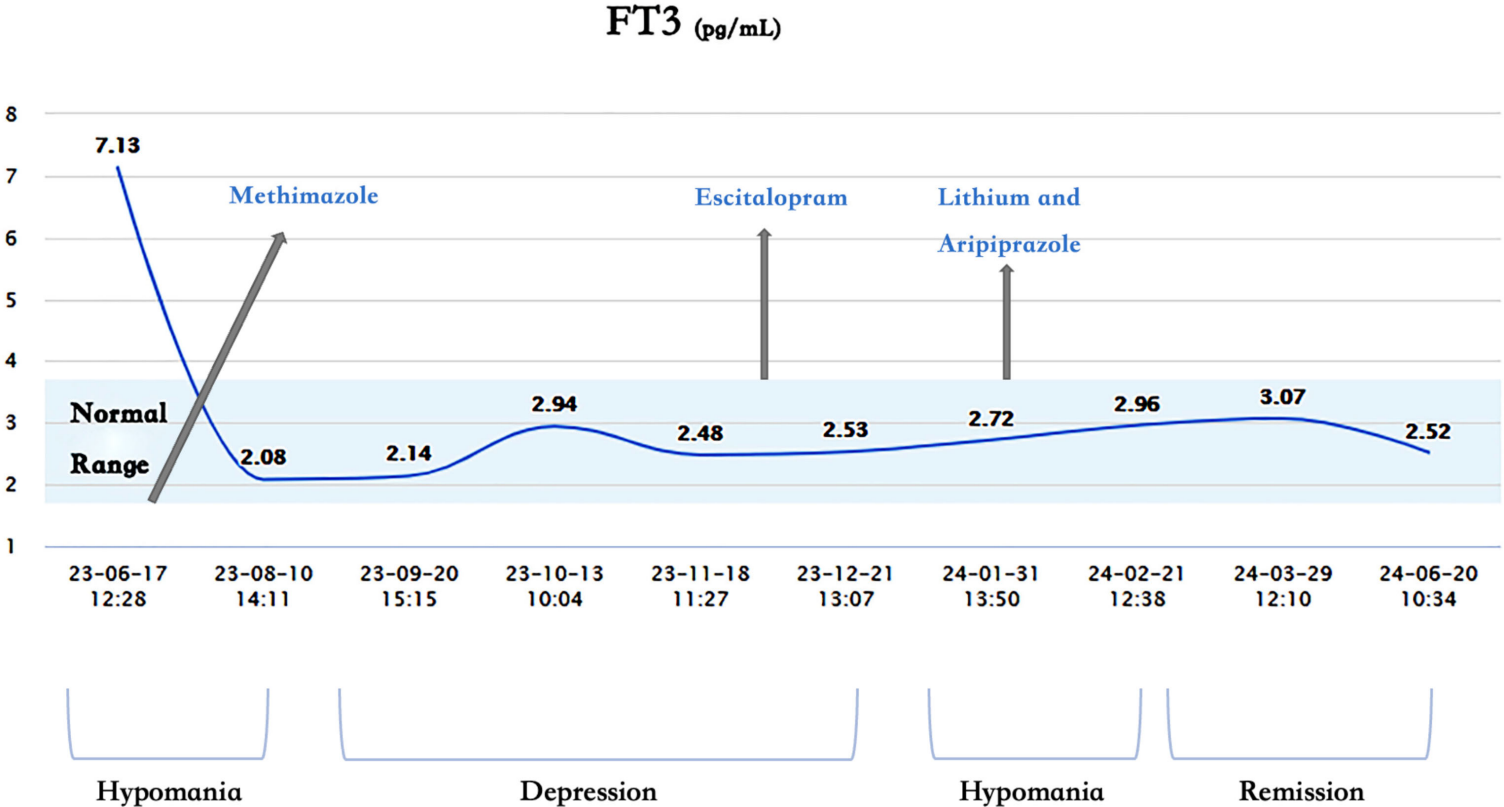
Changes of free triiodothyronine (FT3) and mood.

**Figure 3:**
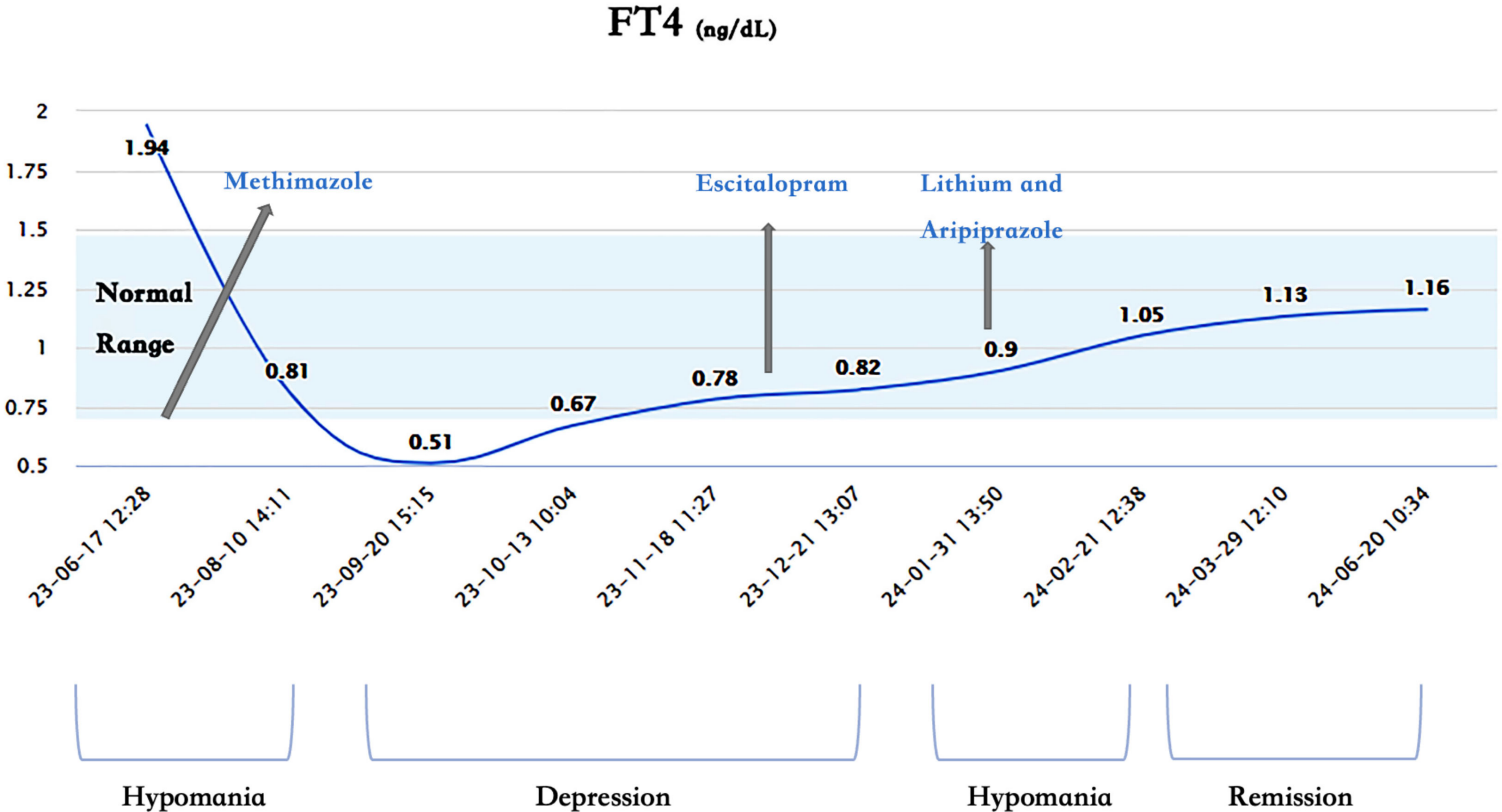
Changes of free tetraiodothyronine (FT4) and mood.

We believe that this patient’s diagnosis should be bipolar disorder co-morbid with hyperthyroidism rather than bipolar disorder due to hyperthyroidism. The reasons are as follows:

1. Lack of Correlation Between Mood and Thyroid Function: The patient’s mood and thyroid function did not show a significant correlation. Her thyroid hormones were in the normal range from 1 month before hospitalization to 5 months after discharge, during which she experienced three periods: depression, hypomania, and remission (**Figures 1**–**3**). Without adjusting methimazole or thyroid hormone medication, her mood was stable during the five-month follow-up after using mood stabilizers, unlike the two episodes of hypomania and one episode of depression in eight months before using mood stabilizers.

2. Hypomania Not Due to Hyperthyroidism: The common emotional and behavioral symptoms seen in patients with hyperthyroidism is anxiety, restlessness, irritability, and emotional lability (4), while mania or hypomania is a multi-symptom syndrome with elevated or irritable mood as the core symptom. According to DSM-5-TR, medical conditions more commonly leading to mania or hypomania include hypercortisolism, multiple sclerosis, stroke, traumatic brain injury, and systemic lupus erythematosus, not hyperthyroidism (5).

3. Depression and Hypothyroidism Not Closely Related:Hypothyroidism, a common side effect of metiomidazole, followed by depressive symptoms. We believe that hypothyroidism may also be a trigger, not a cause. The most important reason is that one month after thyroid function returns to normal, the patient’s mood is still depressive.

4. Existing Obsessive-Compulsive Symptoms Before Hyperthyroidism: The patient had obsessive-compulsive symptoms for several years before the hyperthyroidism diagnosis, and previous annual physicals showed no thyroid function problems. Several clinical features (e.g., mood switches, mood instability, depressive episodes, anxiety, sleep disorders, attention deficits, conduct disorders, and substance use disorders) are associated with a higher risk of developing bipolar spectrum disorder. These symptoms or syndromes may appear years (1.8 to 7.3 years) before an acute manic or hypomanic episode (6). The prevalence of obsessive-compulsive disorder (OCD) in patients with BD is approximately 17%, and OCD is associated with the onset of BD with a hazard ratio of 6.9 (7). OCD may be a key feature in the development of BD.

Although we do not believe it is the cause, hyperthyroidism may have played a role in stimulating the bipolar disorder. A systematic review found that thyroid autoimmunity is an independent risk factor for bipolar disorder (8). Some studies have found a clear association between hyperthyroidism and bipolar disorder (9, 10), while others have suggested that hyperthyroidism may increase the risk of developing bipolar disorder (11, 12), with one study even finding that the correlation between hyperthyroidism and bipolar disorder was the highest of any psychiatric disorder (13). Neurobiological studies have uncovered some mechanisms of thyroid hormone in the brain, providing possible explanations for the interaction between thyroid hormones and emotions (14, 15). However, it is important to note that the above studies were conducted on two disease subjects, rather than two manifestations of a single disease occurrence.

Hypothyroidism was considered to be an etiologic factor for depression, and is also described in DSM-5-TR as a common medical condition leading to depression (5). However, in a systematic review and meta-analysis, the effect size of the association between hypothyroidism and depression was substantially lower than previously hypothesized (16). The psychiatric symptoms of hypothyroidism are most often characterized by slowed mentation, poor concentration, and decreased short-term memory, followed by social withdrawal, psychomotor retardation, depressed mood, and apathy (17).

The age of onset of bipolar disorder is generally low, but the patient in this case did not start until she was 40 years old. One systematic review found that the age of onset of bipolar disorder showed a trinomial distribution: early-onset at an average age of 17.3 years (45% of cases), mid-onset at 26 years (35% of cases) and late-onset at an average age of 41.9 years (20% of cases) (18).

In the context of the biopsychosocial model, the idea of mind-body integration of disease is becoming more emphasized. In this case, the patient developed hypomanic episode after hyperthyroidism and major depressive episode after hypothyroidism, leading to a monistic diagnosis. However, the User’s Guide for the SCID-5-CV Structured Clinical Interview for DSM-5 Disorders states that a close temporal relationship does not necessarily imply a causal relationship on a physiological level. The diagnosis of “…… due to another medical condition” is relatively rare, and co-morbidity between psychiatric disorders and somatic diseases is much more common (19).

From this case and the previous literatures, the relationship between thyroid function and mood was found not to be as strong as previously hypothesized. The relationship between other somatic diseases and psychiatric disorders also requires more researches. When psychiatrists encounter such patients, we need to be more patient to focus on the symptom correlation and more time for observational follow-up. When in doubt, our default assumption should be that the somatic disease is not the cause (i.e., the psychiatric disorder is primary).

## Data Availability

The raw data supporting the conclusions of this article will be made available by the authors, without undue reservation.
